# What would it take to meaningfully attend to ethnicity and race in health research? Learning from a trial intervention development study

**DOI:** 10.1111/1467-9566.13431

**Published:** 2022-01-12

**Authors:** Tanvi Rai, Lisa Hinton, Richard J. McManus, Catherine Pope

**Affiliations:** ^1^ Nuffield Department of Primary Care Health Sciences University of Oxford Oxford UK; ^2^ The Healthcare Improvement Studies Institute University of Cambridge Cambridge UK

**Keywords:** ethnicity, medical sociology, race, racism, randomised controlled trials, social inequality

## Abstract

The lack of ethnic diversity in health research participation is a multi‐dimensional problem. Racism and intersectional disadvantage compel us to use racial and ethnic categories to explore health, but race theorists warn that these can be essentialising and pathologising. Yet, the alternative, the pursuit of colour‐blindness, can render the impact of race and ethnicity on health invisible. This paper describes the attempt to recruit an ethnically diverse sample to inform the development of an intervention for stroke patients. The study revealed deep uncertainties and tensions, which we use to re‐examine our own positionalities and perspectives. We focus on the experiences of researchers and participants to show how ‘usual’ research practices are unwittingly exclusionary and promote ‘methodological whiteness’ (*The British Journal of Sociology*, 2017, 68, S214). Calls for greater diversity in research are frequently made, yet health research remains tainted by the use of problematic epistemological starting points, rendering participation by minoritised people uneasy. Medical sociologists, especially those engaged in clinical trials, have a vital role to play in recalibrating health research to attend to ethnicity and race. This requires us to reflect on our practices, to recognise where we are complicit in replicating social inequalities and to actively engage with communities to produce more inclusive research.

## INTRODUCTION

In this paper, we critically examine an intervention development study we conducted, prior to a large randomised controlled trial (RCT). Our study involved optimisation of a home‐based blood pressure monitoring intervention for people who have had a stroke. Improvements in stroke incidence seen in white populations have not been replicated in racially minoritised groups (Wang et al., 2013), and these groups are poorly represented in RCTs testing the effectiveness of treatments (Wright, 2020). Through this paper, we hope to ignite an important conversation for medical sociologists and those doing health related research, about representation and inclusivity, particularly in the context of RCTs. We focus mainly on the representation of racially minoritised (Gunaratnam, 2013; Selvarajah et al., 2020) groups in trials, but also consider other forms of marginalisation as we contemplate our complicity in propagating methodological whiteness (Bhambra, 2017a, 2017b) via research methods that fail to address material and social disadvantage and discrimination.

## BACKGROUND

Health research in the wealthiest and most research‐active countries of the world has faced a persistent problem of insufficient representation from racially minoritised populations. If trial populations are unrepresentative of actual populations affected by illness, then interventions, treatments, guidance or policy may not be tested on the full range of research beneficiaries (Clark et al., 2019). RCTs are the backbone of whether and how most health interventions are introduced into a health system, and if the methodological foundation of trials is exclusionary, this can become a matter of life and death. In the UK, the INCLUDE guidance introduced by the National Institute of Health Research (NIHR) (NIHR, 2020) seeks to redress this by setting an expectation that clinical research should include groups that have historically been forgotten, ignored or under‐served (Treweek, 2020). Medical sociology has always had a strong focus on health inequalities and the experiences of disadvantaged groups and has some track record (although perhaps not as much as we might like) in exploring the links between race, ethnicity and health (SHI, 2020). Perhaps because of frequent dual‐employment as methodologists and as teachers in medical schools, medical sociologists often collaborate in clinical research, notably in RCTs (Rooshenas et al., 2019) and they often support intervention development or process evaluation. Broadly speaking, their role is to inject a healthy dose of ‘context’ into clinical and health research, so that the social and structural factors affecting health, illness and health behaviours are kept in clear sight, from conception of the original study protocol, right through the duration of the research.

Researching health in ways that account for the influence of race and ethnicity is a slippery business. The conceptualisation of racial differences has an ignominious history, rooted in European imperialism and colonialism: as Roberts so powerfully argues, ‘Race was invented in order to implement racism’ (Roberts, 2021: 17). Race has been discredited as an objective, biological categorisation (Saini, 2019; Yudell et al., 2016), most significantly on completion of the Human Genome Project (NIH, 2003) which found the vast majority of genetic variation exists within racial groups and not between them. Although revealed as a social category, race remains a powerful a determinant of health (Dhairyawan, 2020) and there is fast‐gathering evidence that *racism* not race, acts as a ‘fundamental cause’ (Link & Phelan, 1995) of health inequalities (Bécares et al., 2015; Dess et al., 2019; FitzGerald & Hurst, 2017; Rao et al., 2020). Yet such is the power and endurance of racial and ethnic categories that they continue to be deployed as proxies for a complex combination of ancestry and family history, the intersecting effects of social, economic, political and environmental contexts (Boyd et al., 2020; Saini, 2019; Williams et al., 2019) and racism experienced over lifetimes and generations (Geronimus et al., 2006). Poor theorisation masks intersectionality and nuance and fails to comprehend how multiple contextual disadvantage and structural discrimination linked to racial or ethnic labels engenders poor health (Krieger, 2000; Nazroo et al., 2020).

Pollock (2012) and Smart and Weiner (2018) have argued that all too often racial and ethnic categories are operationalised to measure the incidence of disease among ‘non‐white’ populations, in ways that close down socio‐political questions about what race and ethnicity means (Pollock, 2012; Smart & Weiner, 2018). In the UK, the term *race* implies a ‘biological’ classification, mainly based on skin colour or other morphology, whereas *ethnicity* implies a ‘cultural’ grouping of people with shared history, customs and traditions. The terms are often used interchangeably in academic and scientific writing, although *ethnicity* enables discussions of fixed group‐level characteristics without invoking the distasteful associations with colonial cruelty that *race* has (Williams, 2021).

Alongside a failure to critically engage with these problems of categorisation, another equally problematic silence in health research concerns the fact that racial categories have historically been used to exploit groups for medical experimentation (Washington, 2007). Responses to these unethical practices have led to a kind of ‘performative colour‐blindness’ (Younis & Jadhav, 2020: 620) in some UK‐based research, which ignores how race configures present‐day health inequalities. Far from being an anti‐racist manoeuvre, colour‐blindness enacted as ‘treating everyone the same’ exonerates and so legitimises the present British reality of the relative advantages enjoyed by some racialised groups (i.e. white‐majority populations) over others. We need continued engagement with race precisely for it to be rendered visible, comprehensible and, so, addressable. By adopting a ‘post‐racial’ stance, we willingly blind ourselves to the myriad ways in which we normalise exclusion in research practice. In the UK, for example, if we design and enact RCTs that recruit from white groups despite higher prevalence of disease in racially minoritised groups, we render invisible the very real relationships of inequality between these groups.

Recognising that racial and ethnic categories are ‘socially constructed, relational and socially located’, Gunaratnam (2003)—quoting Hall (1996)—argues compellingly that these terms should be used in a deconstructed way: they should be considered ‘under erasure’, as no longer ‘good to think with’ but yet to be replaced by something better (Gunaratnam, 2003: 31, Hall, 1996). Following her lead, it appears that, for the time being, race and ethnicity are necessary, if imperfect, categories that allow us to consider and respond to patterns of social, educational, economic, political and health inequalities and disadvantage, and we use them with these cautions in mind.

We also use Bhambra's concept of ‘methodological whiteness’: ‘a way of reflecting on the world that fails to acknowledge the role played by race in the very structuring of that world, and of the ways in which knowledge is constructed and legitimated within it’ (Bhambra, 2017a). Echoing historians of postcolonial theory who study problematic normalisations (Chakrabarty, 2000), Bhambra argues that in white‐majority countries like the UK, the white perspective is normalised as universal, pushing all other (non‐white) perspectives to the periphery. She challenges us to understand race as an organising process and epistemological frame and to think harder about what ethnicity *means* in research. In taking up this challenge in the context of health research, we see how easily, in addition to biological claims, ‘cultural differences’ are marshalled as explanations for variations in health status for non‐white ethnic groups. For non‐white people, collectivised, ‘culturally competent’ explanations (Metzl & Hansen, 2014) are used to explain nearly everything from dietary choices to sexual practices to medication adherence. Meanwhile, although ‘whiteness’ has been the subject of some reflection within the social sciences (see for example, McIntosh (1989) and Byrne (2006)), medical and health researchers seldom enrol the ‘collective’ white cultural experience to explain white people's health outcomes in the ways they do for non‐white ethnic groups. Taken together, these practices and the shameful history of scientific racism reveal the epistemic continuity between Eurocentric approaches used to justify colonialism, slavery and unethical medical experimentation, and the normalisation of research practices which ‘other’ non‐white people.

For this paper, we take these challenges on board and attempt to practice ‘epistemic disobedience’ (Mignolo, 2009: 160). This entails a conscious break from the ‘illusion of the zero point epistemology’ (Mignolo, 2009), a system of classifying people, problems and projects in ways that favour those in power. We explore how normative and taken‐for‐granted research methods found in RCTs beget ‘normal’ data, which are then privileged as legitimate knowledge. Through this sometimes uncomfortable, reflexive exercise, we invite other medical sociologists and health researchers, especially those engaged in trial research, to reflect on their own methodological practice.

## METHODS

This paper is based on qualitative research conducted from September 2018 to June 2019 as part of a project to develop a complex intervention for home‐based blood pressure management for people who have had a stroke. Details of the project are published elsewhere (Rai, Morton, et al., 2021).

Data sources used are listed below:

- 24 think‐aloud face‐to‐face interviews seeking feedback about the trial materials
- 11 retrospective interviews with participants who had used the prototype intervention
- Four focus groups with healthcare professionals
- Researcher field notes from 10 conversations (in person and telephone) with community leaders and stakeholder contacts, and from community visits to churches, mosques and stroke support groups.

In addition, the lead researcher's (TR) records of study team conversations, email exchanges within the team and with charity partners and the PPI co‐investigators have informed the arguments presented here.

Participants’ ethnicities were described by TR using her judgement based on visual cues and the interview material, and informal discussions with key contacts/introducers and participants themselves about their ethnic identity, family background, etc. (this was typically discussed in the preamble to the interviews).

Participant characteristics are detailed in Table 1.

**TABLE 1:** Characteristics of 35 participants in the intervention development study

	Recruitment via:		Totals
	General practices	Community networks	
Gender			
Male	13	9	22
Female	10	3	13
Age			
35–50	1	3	4
51–65	5	5	10
66–75	6	2	8
>75	11	2	13
Ethnicity			
White British	20	3	23
White Other	3	1	4
Black African	0	2	2
Black Caribbean	0	1	1
Mixed ethnicity	0	1	1
South Asian	0	2	2
East Asian	0	2	2

Given our focus on race/ethnicity and complicity, it seems vital that we reflect on our own positionality, and, on our racial and ethnic identities. Three authors of this paper are white, one (RM) is an academic and GP, LH and CP are social scientists with expertise in qualitative research. The lead researcher (TR), who collected the bulk of the data presented here, is an experienced mixed‐methods researcher and is Indian. We want at the outset to challenge the inherent racism of definitions based on skin colour, and yet already, so early in the paper, we find ourselves performing whiteness, and reaching for its binary, ‘non‐white’? Or Black? Or perhaps Brown? This is discomforting. We therefore offer an extract from reflexive notes made by TR describing her identity in her own words, to add nuance:

I am an Indian migrant who came to the UK 23 years ago on an academic scholarship. Although I have experienced some racism in the UK, I have also experienced enormous privilege, first as an upper caste, Hindu, English‐speaking person in India, and then in the UK, studying and working at leading academic institutions and being partner to a White British man. Over time, I have learned the cultures, codes and nuances of language used within predominantly white spaces, and can often “pass” as more familiar than foreign. The feeling of “otherness” never goes, indeed it is sometimes reinforced in particular social interactions. Within the research team, I find the description of “outsider within”, borrowed from critical race theory (Ford & Airhihenbuwa, 2010: S31), helpful to explain my feelings. Being the “other” can happen across many intersecting dimensions, not just across ethnicity or race. Like everyone, I straddle multiple social worlds and regularly adjust my social identity according to my surroundings.

We return to our reflections on researcher identity later in this paper but emphasise that while we have worked together on the arguments presented, TR is closest to the data described here. To avoid confusion, we use the third person when referring to the ethnographic fieldwork and first person when referring to our current reflexive state. The affective experiences of research are TR's. The initial phase of the analysis used ‘typical’ qualitative, iterative, thematic analysis to optimise the intervention and trial processes (Rai, Morton, et al., 2021). For the second phase, underpinning the arguments for this paper, we revisited the larger corpus of data, re‐interrogating the transcripts and drawing more heavily on TR's reflexive field notes. We scrutinised and discussed data excerpts and themes as a team, and what began as a process of informing the development of an intervention and a trial became an examination of uncertainties and tensions pertaining to race and ethnicity in research practice.

## FINDINGS

The analysis and discussion below is organised into three themes. First, we describe the efforts we made to diversify the sample for our study. Second, we examine the participants' responses to our research processes and materials; and finally, we reflect on positionality, identity, intersectionality and biases, as the basis for a broader discussion about what medical sociologists and other researchers can do to attend in more meaningful ways to ethnicity and race in health research.

### The process and practice of patient recruitment

Trial timelines and metrics used to measure success often prioritise fast recruitment, and procedures are streamlined, highly routinised and designed to be scaled up rapidly. Although ours was a pre‐trial development study, it was intended to mimic these trial recruitment processes.

Following ‘normal’ procedures for smaller studies based in primary care, General Practices within a single region (in South‐East England) were invited to take part. Most that volunteered were already ‘research active’, and their engagement was scaffolded by prior collaborations with the research team. Only one Practice scored high on measures of social and economic deprivation. Using these ‘easy’, well‐trodden recruitment pathways is efficient and likely to generate swift results. Evidently, the tight deadlines set by funders offer validation for these choices, but can hardwire the repeated exclusion of groups that other less research‐savvy sites are well positioned to provide (Rai, Dixon, et al., 2021). In pursuit of a more diverse sample, TR invited three additional General Practices known to serve ethnically and economically diverse populations, but all three declined, citing heavy workloads and a lack of research capacity. This was disappointing but helps to illustrate how, even at the site‐selection stage, structural inequities shape and striate research: Practices in high‐deprivation areas are often too under‐staffed and under‐resourced to support research.

This Practice‐based recruitment captured predominantly more educated and middle class participants, who were white. This troubled TR and LH, who were aware that people from racially minoritised and socioeconomically disadvantaged groups had more strokes, at younger ages, of greater severity, with worse outcomes and with increased risk of reoccurrence (The Stroke Association, 2018), and thus had more to gain from the intervention. Although noted as a clear deviation from normal practice, and with the explicit instruction to keep to data collection deadlines, TR (with the support of LH) successfully appealed to the wider team for ‘permission’ to explore methods to widen the diversity of the sample.

She contacted local Pastors and Imams serving large congregations of people of African, Caribbean and South Asian ancestry. A few phone calls later, she had invitations to attend services at two mosques and two churches to speak about the research.

Following discussion with the religious heads, for the Sunday morning church visits, TR wore ‘Sunday best’ western clothing, and for the Friday mosque visits, she dressed in salwar‐kameez and a head scarf. The church congregations were majority Black. On being invited on stage by the Pastor, TR did a presentation about the study, including a slide about poor representation of Black people in research and the disproportionate stroke risk for them (appealing to their ‘ethico‐racial imperative’, see Williams (2021)). She also participated in church activities for over two hours, listening to speeches, singing and bible study discussions, after which she set up a ‘research table’ with blood pressure monitors and study leaflets. At both mosques, the congregation was mostly South Asian and some Black people. Being female, TR was unable to go into the men's prayer halls but provided the Imams with a script to introduce the study. The women's prayer halls were less busy so TR set up her research table just outside the men's hall. Many of the working age men rushed past, sometimes picking up a study leaflet. Those with more time came over to find out more. The fact that TR spoke Urdu allowed her to speak with many congregants, especially older people less comfortable speaking in English. Between 10 and 40 people at each mosque and church meeting took the opportunity to have their blood pressure taken and discuss the study. Several wanted to take part, but only four met the inclusion criteria (having had a previous stroke) and were recruited to the study.

Additional community‐based recruitment occurred by visiting stroke clubs and by liaising with the research funders’ support staff based in high‐deprivation areas.

The above description provides just a small illustration of how *different* our recruitment methods had to be from the normalised process of using automated mass mail‐outs in order to achieve more demographic diversity. Attempting epistemic disobedience (Mignolo, 2009) even on this (relatively) small scale had required courage (to convince the team), and a broader set of resources than those listed in the original recruitment plan. These methods were time‐intensive, highly interactive and relied heavily on the sociality of TR, who occupied a dual identity as a researcher from an ‘elite’ university, but also someone with direct experience of exclusion across racial/cultural divides. Empowered by the novelty of these atypical recruitment methods and spaces, she relished ‘mucking in’ during these sessions, finding ways to demystify the research processes and connect to groups who are so often ignored in trial research. Her careful choice of appropriate clothing was designed to signal respect and establish rapport. TR noted that these choices felt like a political act; in particular, she found it thrilling to be able to wear her salwar kameezes during fieldwork without fear of encountering prejudice or hostility, or being doubted as a credible academic. Wearing these clothes for the mosque visit was in fact an asset and offered the chance to champion an alternative ‘new look’ for academic researchers.

Some research has a deliberate focus on non‐white populations (Wood et al., 2012) but this trial was planned as ‘a normal trial’, so while the team had an ‘in principle’ commitment to diversity and inclusion, the triallists had not anticipated enfolding ethnically diverse recruitment into this study. It is only in retrospect that we have begun to understand how the ‘usual’ research practices we ‘normally’ follow, ignore or silence the voices and experience of some and privilege others, and how positionality, identity and presentation (and even clothing) impact on trial methods. As Younis and Jadhav (2020) suggest, a consequence of performative colour‐blindness is that some people are repeatedly omitted from mainstream discourse.

Having discussed our recruitment practices, we now turn our attention to how different people responded to being asked to contribute to the research.

### Participants' responses to being asked to contribute to intervention development

In this section, we illustrate how some research practices may be experienced differently by participants who are not white and/or middle class. The first example is an encounter with Mr F, who is an elderly, Asian man and retired taxi driver. He lives with his wife and rents out a couple of rooms in his house to lodgers. TR's field diary describes their meeting as follows:

When I arrived at the house, I was offered tea by Mrs F. We quickly established our shared ethnic heritage and this allowed me to choose “desi”‐style tea (boiled with milk and ginger) rather than “English” tea. While I was getting the materials out I chatted with Mr F in Urdu, and then moved to some opening questions about his stroke experience, speaking partly in Urdu, partly in English. The think‐aloud section of the intervention development required participants to read the materials, such as the patient support booklet, and verbalise responses to the content in real time. This created a noticeably awkward linguistic turn. Where the conversation had been smooth and flowing, Mr F now became stiff. He sat more erect and concentrated on reading aloud. His English fluency was not perfect; he misread and missed some words and appeared to struggle with the amount of information on each page.

Having trouble reading may of course be partly due to older age and language processing deficits following stroke. Moreover, in common with several older participants, especially those from disadvantaged groups, some parts of the study information booklet made little sense to Mr F. In response to questions about text messaging or using an app, Mr F produced his smart phone from a cupboard and he turned it on to show it to TR, explaining that he only used it for phone calls. It was therefore unsurprising that he found it difficult to comment on this part of the study design.

The incongruousness of the study design and indeed the intervention itself in the context of participants from less privileged backgrounds also surfaced with a Caribbean participant, Mr M, who lives with his wife and adult daughter in a high‐deprivation neighbourhood and runs a youth support centre:

When I asked Mr M to read the patient booklet aloud he obliged, although this seemed to tire him, and at the end of every page he stopped to ask me if he was doing okay, and if I wanted him to carry on. I felt guilty for making him do this task. It was slow and laboured, and felt as if I was ‘testing’ his reading ability. Every time I asked him how he found the content, the layout and the phrasing, he repeated that it was fine. When I related my discomfort to a colleague in the wider study team they responded with: “He said ‘It's fine’ so he found it acceptable, and that's great.”

Having already pushed the boundaries of normal trial practice by engaging in ‘additional’ recruitment TR decided not to pursue her concerns about taking this ‘it's fine’ response as a measure of acceptability. This ‘falling into line’ was disappointing for her, but was driven by fear—she had already experienced some resistance within the wider team to her recruitment methods, and as a more junior, and contractually precarious and only non‐white member of the team, aware of power and hierarchy, she felt unable to speak again on this issue. It was only in the writing of this paper, which unearthed some quite uncomfortable discussions among the writing team, that this episode was revealed as another way that trial epistemology and practice, combined with racialised and gendered precarity of research staff within academia, encourages complicity with methodological whiteness. In essence, although the remit of the intervention development study was to assess acceptability, it only permitted binarised responses. There was no room for the nuanced concerns raised by TR, and we see now that in taking Mr M's ‘it's fine’ response at face value (despite strongly suspecting that he was *not* fine) the research team may have succumbed to a degree of superficial tokenism in order to validate and press on with the research.

Far from raising the alarm about inequitable research practice, we, as medical sociologists embedded in trial research, actively produced ‘trial‐compatible findings’, despite seeing clearly the uneven ways in which the study was received. In writing this paper, we have begun to comprehend how our complicity comes from inaction and a failure to challenge trial‐normative agendas. The power structures within the trial team, pressure from funding timelines, and the lower status of medical sociologists acting as ‘handmaidens’ supporting and validating the *main* objective of setting up the medical trial enforce epistemic ‘obedience’ (Mignolo, 2009): in our case, it focussed our attention on the task of ‘optimising’ the intervention as quickly as possible, even if this silenced important findings in the process.

In contrast to Mr F and Mr M, the majority of people recruited via GPs talked comfortably and at length, some used medical terminology, and related interactions with doctors and health systems that sounded responsive and congenial. They found it easy to apply the proposed intervention to their personal circumstances and to give feedback. Those with negative opinions did not hesitate to share them, for example ‘*It is outrageous to expect older people to have such a low BP*’ or ‘*With respect*, *this section hasn't been put together very well’*. In contrast to Mr F and Mr M (and some *white* participants recruited via community groups in deprived settings), comparatively little time was required to build trust or explain the research to them.

If we only use easy routes to obtain new knowledge, we bias what we privilege as *worth knowing*. Moreover, when we deliberately ignore new knowledge that potentially introduces grit into the smooth running trial machine we become complicit in the reproduction of inequity. If we continue to maintain the deception that if something is worth knowing, it will be straightforward to obtain, and continue to abstain from properly engaging with the methodological politics of how knowledge is constituted, we neglect our ethical responsibility towards our participants.

### Researcher identity, positionality and complicity

We now explore our reflexive analysis in response to the intervention study data. We identified two sources of obvious discomfort. The first was an emotional response to participants’ responses to the research tasks: our encounters with participants from racially minoritised groups *felt* more exploitative than encounters with white respondents. Second, in the writing of this paper, we surfaced differences of opinion and understanding within the writing team, which forced us to think harder about researcher identity and responsibility.

#### Exploitation

Research necessarily *exploits* people in the sense of ‘making use of’ and ‘deriving benefit from’ their participation. However, the secondary, morally‐informed linguistic usage of this word denotes activity that can be considered unfair or underhand. The racially minoritised participants appeared to us more precarious than the white participants recruited via General Practices, partly because their employment and housing circumstances suggested higher levels of insecurity. Reflecting the statistics, they had more post‐stroke disabilities; some were of working age but were incapacitated and therefore had reduced or lost incomes, and this had precipitated further ill‐health, including depression.

Ms A, from Southeast Asia, received the study training (which involved taking and submitting blood pressure readings over a week), but only managed to submit one reading before giving up. Her stroke had left her paralysed from the chest down; she had young children, and her husband, the sole breadwinner, worked long shifts in a factory. When TR revisited her, she was very apologetic about not completing the tasks. She praised the monitor and the intervention, framing non‐participation as her fault:

There were so many things going on, kids' exams and also myself, so there's so many things…. I was also emotionally a little bit low because, I can't really keep up with everything, I could have done better if I wasn't in that situation, all of that, …and by the time I, I sort of remember it, it was probably already too late and I’m thinking, “Okay I'll start the next day make sure in the morning”, in the morning sometimes, I'm in a lot of pain, and I took a lot of pain relief and just feeling a bit sort of, shit. Yeah, so that was the reason, but, this machine [BP monitor] is really good.

Ms A's social and physical disadvantages prevented participation: Her life did not ‘fit’ with the research timeline and requirements. Later, she was keen to re‐engage, but it was too late: The study had moved on without her.

These data raise questions for us. We opened space to get diverse perspectives, albeit with some persuasion, yet with no capacity to actually respond appropriately to the needs and experiences of minoritised people. Constricted by the rigid data collection tools we had chosen to employ, we never discovered what Mr F and Mr M *really* thought about the intervention. Nor did we explore how parts of it might be confusing or alienating, and thus, we missed crucial opportunities to make the design accessible to a much wider social demographic. The temporality of trial projectification (determined by the need to present a *competitive* set of discrete and time‐bound ‘work packages’ to funders) is raced in such a way that it systematically excludes socially disadvantaged people who are less able to meet research expectations and timelines, even when those very groups might have the *most* to benefit from the research. On reflection, we now fear that by planning only for ‘representation’ and not being able to respond suitably to how minoritised people really felt about the intervention, we may have exploited them. We were able claim success at achieving a *diverse* sample that included Asian and Black participants, but this was somewhat tokenistic. We shied away from our responsibility to use insights from these participants to interrogate, let alone disrupt the conceptual and methodological framing of the trial and its underlying (exclusionary) assumptions.

#### Silencing participants and researchers

Study protocols and timelines also sometimes limited receptiveness to unexpected insights. For example, during the focus group at the Practice serving an economically deprived and ethnically diverse area, one (white, female) GP complained that the intervention did not accommodate patients fasting for Ramadan. Her concerns were shared with the research team, but because this issue had not been raised by other Practices (which had fewer Muslim patients), and again because of pressing research deadlines, the team decided that responding to this comment was not essential to the success of the trial. While the number of fasting patients may be too small to have a statistically significant effect in the trial, this illustrates how feedback relating to minoritised groups can be lost.

We described earlier how researchers may be silenced within a hierarchical study team, but it may also occur within the micro politics of researcher–participant interactions (Bhavnani, 1993). As mentioned, TR is a ‘brown’ researcher and many of the study participants were white. One set of field notes, following an interview with a white couple, reports:

Interview with couple, Mr and Mrs R: We started with the usual conversation about his stroke and how often he sees his GP. They were complaining about how difficult it is to get an appointment and then *she complained about how so many of the GPs are women, who work part‐time […] so it's difficult to see the same GP. Then she said that the first GP they offered her, she couldn't even pronounce the name so she asked specifically for an “English GP” and they were good about giving her that*. I got the feeling she would have said more but maybe some self‐awareness crept in […]

Such interactional irritations are not unique. Many white researchers experience ‘awkward’ moments conducting interviews. However, for racially minoritised researchers, interpersonally experienced racism is an additional burden, and informal conversations with other non‐white researchers suggest these experiences are not unusual. In writing this paper, we realised how rarely we create space to acknowledge such experiences, yet if these aggressions are not addressed, then we are all complicit in preserving historically racialised inequalities, even among our academic peers. Some have suggested that having more diversity in the academy is likely to produce more inclusive research (Krieger et al., 2021). The learning from writing this paper provoked welcome discussions among research leaders and senior academics in our department about supporting non‐white researchers better, within research groups and during fieldwork, and how we might better reward the contributions of bridge builders between historically ‘white’ institutions and racially minoritised groups.

## DISCUSSION

In this paper, we have attempted to shine an uncomfortably bright light on our own research practice to explore how epistemic and methodological practices become complicit in the reproduction of inequity. The planned intervention for the RCT was designed to reduce the risk of a repeat stroke, and minoritised groups are known to be at higher risk for this because of their intersectional disadvantages (Boyd et al., 2020; Krieger, 2000). While these populations may be a minority within the total numbers of strokes each year, having severe strokes at younger ages can have catastrophic long‐term effects for them and their families, so there is a strong case for ensuring they are included in stroke research.

Calls for greater diversity in research have been amplified by recent racial justice and equality movements. The intervention development study began using ‘standard’ recruitment procedures, which elicited many positive responses, but did not adequately reflect racially minoritised or socioeconomically deprived groups. The additional community‐based recruitment activities we undertook were atypical, but succeeded in engaging a more diverse sample.

We believe the participation burden on minoritised participants was greater, both linguistically and as a practical accomplishment, compared to white and/or middle class respondents, because of the tools and timelines we worked with. Moreover, our primary commitment to facilitate timely optimisation of the intervention in the lead up to the trial prevented us from privileging some responses as legitimate findings, to be unpacked and worked into the intervention design (e.g. Mr M's unease when reading the study booklet or Ms A finding the intervention unworkable).

Hence, a word of caution here. We need to go further than mere representation of minoritised groups. Without adequately reconfiguring our methods to respect and protect the vulnerable, we run the risk of exploiting or exposing them further. We need a more responsive research process, which is contextually grounded so that we do not just elicit responses, but also have the time and space to incorporate them into the outcomes of research.

Our paper suggests that institutional and research funding imperatives, and ‘normal science’ get in the way of more thoughtful, inclusive inquiry. In large RCTs, a complicity with maintaining methodological whiteness is baked into the system in multiple ways, right from the enrolment of favoured research sites, to routinised methods and procedures that filter out participants who lack literacy, English fluency and digital proficiency, and research cultures and timelines that discourage the collection and analysis of data that might challenge the basic assumptions upon which a trial is built.

These complicities are deep inside our research practices. TR, despite not having previously engaged too deeply with how her own racially minoritised position affected the research context, found herself raising questions about race and ethnicity in meetings with a large, all‐white research team, and with her white co‐authors here. Such conversations about widening the research lens are uncomfortable but not new. Epstein (2009), Healy (2003) and Perez (2019), for example, have consistently critiqued the systematic erasure of women from medical research. The presence of TR as an ‘outsider within’ (Ford & Airhihenbuwa, 2010) pushed the study team to try something different and provoked the three white co‐authors to reflect more deeply on anti‐racist praxis in health research. The more senior members of this authorial team have also, in examining this work, become painfully aware that participant recruitment, and by extension work to ‘diversify’ a sample often falls to early career researchers, who themselves may be minoritised in some form and this requires, and should compel, the hierarchically more powerful to speak out. This notwithstanding, we also recognise from our own experience, that as long as research funders impose short timeframes on the delivery of RCTs (and any work nested within them), racialised disparities in research participation may be a problem whose solution lies well beyond the acts of individuals.

Medical sociologists have long critiqued the positivist framing of much medical research. Few would argue that research is neutral and value‐free, but we have perhaps been too careful to separate scholarship and social justice. WEB Du Bois (Du Bois, 1903) advocated that the former should be used to interrogate, challenge and change societal ills and not to replicate inequities. With the growth of implementation science and process evaluation, medical sociologists are increasingly engaged with RCT research, and in these settings (but arguably also in other types of research), they have a potentially pivotal role in shaping the science (Rooshenas et al., 2019). This work is not neutral. Returning to our earlier call for epistemic disobedience (Mignolo, 2009), we encourage all medical sociologists embedded in clinical research to reject their problematic complicities with trial methodology and instead leverage their proximity to this research to take the lead against racialised inequities in medical research. Our study itself was insufficiently disobedient, but writing this paper is a renewed attempt at this, and we hope our retrospective critical evaluation will be instructive and emboldening to others in similar roles who recognise the situations and experiences we have described.

Participation in research invokes notions of citizenship and community responsibility, but for people who have experienced racism and discrimination in many aspects of daily life, equality of participation is more problematic. The expectation that all participants will engage with the study topic, abstracted from and uninfluenced by, other more sinister interactions they might have with other institutional machinery betrays the persistent naiveté of researchers and the insularity of the organisations that undertake research. Consider, for example, how the UK Home Office ‘hostile environment’ policies and contemporary racism within the health service (Fitzgerald et al., 2020; Younis & Jadhav, 2020) might affect these very population groups. We need to acknowledge that cultural incomprehension across groups is often reciprocal—it is not that ‘they’ are different and ‘we’ are not, ‘they’ find ‘us’ different too! Lack of trust is often marshalled as explanation for poor participation by minoritised groups in research (Hussain‐Gambles et al., 2004), but we must recognise that these groups are not *innately* distrustful but may be responding to a long, painful, and continuing history of racism and discrimination, including in the delivery of health care and research, which undermine their status as equals in society. Our foray into this work has confirmed that it is ‘less about what patients have failed to feel and more about what systems have failed to do’ (Boyd et al., 2020). Researchers within contemporary medicine *and* sociology need to work harder and in more explicit ways to disrupt the problematic complicities identified here. Through writing this paper, we can see that there may be many opportunities for productive complicities between triallists, who want robust and generalisable trial results and medical sociologists, who have the skills to advise on trial design to facilitate this. The challenge is not just to shift ‘normal science’ by inviting and enabling diverse groups to participate, but to address more directly the structural and agentic barriers that have excluded them from the conversation so far (Williams et al., 2020). The first step in this journey is admitting that we can and should do better.

## AUTHOR CONTRIBUTION

TR, LH, RM and CP took part in conceptualisation, analysis, writing, review and editing of this paper. RM and LH were responsible for funding acquisition for the intervention development study, and with TR, they shaped the methodology and did the original analysis. TR collected the qualitative data, LH supervised this qualitative component of the study and CP worked closely with TR to get this paper written during the pandemic. TR deserves all the credit for making us have this conversation.

## Data Availability

The data that support the findings of this study are available on request from the corresponding author. The data are not publicly available due to privacy or ethical restrictions.

## References

[shil13431-bib-0001] Bécares, L. , Nazroo, J. , & Kelly, Y. (2015). A longitudinal examination of maternal, family, and area‐level experiences of racism on children's socioemotional development: Patterns and possible explanations. Social Science & Medicine, 142, 128–135. 26301485 10.1016/j.socscimed.2015.08.025

[shil13431-bib-0002] Bhambra, G. (2017a). Why are the white working classes still being held responsible for Brexit and Trump? https://blogs.lse.ac.uk/brexit/2017/11/10/why‐are‐the‐white‐working‐classes‐still‐being‐held‐responsible‐for‐brexit‐and‐trump/

[shil13431-bib-0003] Bhambra, G. K. (2017b). Brexit, Trump, and ‘methodological whiteness’: On the misrecognition of race and class. The British Journal of Sociology, 68, S214–S232. 29114873 10.1111/1468-4446.12317

[shil13431-bib-0004] Bhavnani, K.‐K. (1993). Tracing the contours: Feminist research and feminist objectivity. Women's Studies International Forum, 16, 95–104.

[shil13431-bib-0005] Boyd, R. , Lindo, E. G. , Weeks, L. D. , & Mclemore, M. R. (2020). On racism: A new standard for publishing on racial health inequities. Health Affairs Blog [online]. 10.1377/hblog20200630.939347/full/

[shil13431-bib-0006] Byrne, B. (2006). White lives: The interplay of ‘race’, class, and gender in everyday life. Routledge.

[shil13431-bib-0007] Chakrabarty, D. (2000). Provincializing Europe: Postcolonial thought and historical difference ‐ New ed. Princeton University Press.

[shil13431-bib-0023] Clark, L. T. , Watkins, L. , Piña, I. L. , Elmer, M. , Akinboboye, O. , Gorham, M. , Jamerson, B. , McCullough, C. , Pierre, C. , Polis, A. B. , Puckrein, G. , & Regnante, J. M. (2019). Increasing Diversity in Clinical Trials: Overcoming Critical Barriers, Current Problems in Cardiology, 44(5), 148–172. 10.1016/j.cpcardiol.2018.11.002 30545650

[shil13431-bib-0008] Dess, R. T. , Hartman, H. E. , Mahal, B. A. , Soni, P. D. , Jackson, W. C. , Cooperberg, M. R. , Amling, C. L. , Aronson, W. J. , Kane, C. J. , Terris, M. K. , Zumsteg, Z. S. , Butler, S. , Osborne, J. R. , Morgan, T. M. , Mehra, R. , Salami, S. S. , Kishan, A. U. , Wang, C. , Schaeffer, E. M. , … Spratt, D. E. (2019). Association of black race with prostate cancer‐specific and other‐cause mortality. JAMA Oncology, 5, 975–983. 31120534 10.1001/jamaoncol.2019.0826 PMC6547116

[shil13431-bib-0009] Dhairyawan, R. (2020). Evaluating values. BMJ Leader [Online]. https://blogs.bmj.com/bmjleader/2020/06/15/evaluating‐values‐by‐rageshri‐dhairyawan/

[shil13431-bib-0010] Du Bois, W. E. B. (1903). The souls of black folk. OUP.

[shil13431-bib-0011] Epstein, S. (2009). Inclusion: The politics of difference in medical research. University of Chicago Press.

[shil13431-bib-0012] Fitzgerald, C. , & Hurst, S. (2017). Implicit bias in healthcare professionals: A systematic review. BMC Med Ethics, 18, 19. 28249596 10.1186/s12910-017-0179-8 PMC5333436

[shil13431-bib-0013] Fitzgerald, D. , Hinterberger, A. , Narayan, J. , & Williams, R. (2020). Brexit as heredity redux: Imperialism, biomedicine and the NHS in Britain. The Sociological Review, 68, 1161–1178.

[shil13431-bib-0014] Ford, C. L. , & Airhihenbuwa, C. O. (2010). Critical Race Theory, race equity, and public health: Toward antiracism praxis. American Journal of Public Health, 100(Suppl. 1), S30–S35. 20147679 10.2105/AJPH.2009.171058 PMC2837428

[shil13431-bib-0015] Geronimus, A. T. , Hicken, M. , Keene, D. , & Bound, J. (2006). “Weathering” and Age Patterns of Allostatic Load Scores Among Blacks and Whites in the United States. American Journal of Public Health, 96, 826–833. 16380565 10.2105/AJPH.2004.060749 PMC1470581

[shil13431-bib-0016] Gunaratnam, Y. (2003). Researching race and ethnicity: Methods, knowledge and power. Sage.

[shil13431-bib-0017] Gunaratnam, Y. (2013). Race GPS: No right turn ahead [Online]. https://mediadiversified.org/2013/08/20/race‐gps‐no‐right‐turn‐ahead/

[shil13431-bib-0018] Hall, S. (1996). Introduction: Who needs identity? In S. Hall , & P. Du Gay (Eds.), Questions of cultural identity (pp. 1–17). SAGE Publications Ltd.

[shil13431-bib-0019] Healy, B. (2003). Challenging sameness: Women in clinical trials. National Institutes of Health, Office of Research on Women's Health.

[shil13431-bib-0020] Hussain‐Gambles, M. , Atkin, K. , & Leese, B. (2004). Why ethnic minority groups are under‐represented in clinical trials: A review of the literature. Health and Social Care in the Community, 12, 382–388. 15373816 10.1111/j.1365-2524.2004.00507.x

[shil13431-bib-0021] Krieger, N. (2000). Discrimination and health. In L. Berkman , & I. Kawachi (Eds.), Social epidemiology (pp. 36–75). Oxford University Press.

[shil13431-bib-0022] Krieger, N. , Boyd, R. , De Maio, F. , & Maybank, A. (2021). Medicine's privileged gatekeepers: Producing harmful ignorance about racism and health. Health Affairs Blog [Online]. 10.1377/hblog20210415.305480/full/

[shil13431-bib-0024] Link, B. G. , & Phelan, J. (1995). Social conditions as fundamental causes of disease. Journal of Health and Social Behavior, Spec No, 80‐94. 7560851

[shil13431-bib-0025] Mcintosh, P. (1989). White privilege: Unpacking the invisible knapsack. Peace and Freedom Magazine, A Publication of the Women's International League for Peace and Freedom, Philadelphia, PA. July/August, 10–12. https://nationalseedproject.org/Key‐SEED‐Texts/white‐privilege‐unpacking‐the‐invisible‐knapsack

[shil13431-bib-0026] Metzl, J. M. , & Hansen, H. (2014). Structural competency: Theorizing a new medical engagement with stigma and inequality. Social Science & Medicine, 103, 126–133. 24507917 10.1016/j.socscimed.2013.06.032 PMC4269606

[shil13431-bib-0027] Mignolo, W. D. (2009). Epistemic disobedience, independent thought and decolonial freedom. Theory, Culture & Society, 26, 159–181.

[shil13431-bib-0028] Nazroo, J. Y. , Bhui, K. S. , & Rhodes, J. (2020). Where next for understanding race/ethnic inequalities in severe mental illness? Structural, interpersonal and institutional racism. Sociology of Health & Illness, 42, 262–276. 31562655 10.1111/1467-9566.13001 PMC7028120

[shil13431-bib-0029] NIH (2003). The human genome project [Online]. NIH National Human Genome Research Institute. https://www.genome.gov/human‐genome‐project

[shil13431-bib-0030] NIHR (2020). Improving inclusion of under‐served groups in clinical research: Guidance from INCLUDE project [Online]. NIHR. https://www.nihr.ac.uk/documents/improving‐inclusion‐of‐under‐served‐groups‐in‐clinical‐research‐guidance‐from‐include‐project/25435

[shil13431-bib-0031] Perez, C. C. (2019). Invisible Women: Exposing data bias in a world designed for men. Vintage. 10.3399/bjgp20X709745 PMC7194768 32354824

[shil13431-bib-0032] Pollock, A. (2012). Medicating race: Heart disease and durable preoccupations with difference. Duke University Press.

[shil13431-bib-0033] Rai, T. , Dixon, S. , & Ziebland, S. (2021). Shifting research culture to address the mismatch between where trials recruit and where populations with the most disease live: A qualitative study. BMC Medical Research Methodology, 21, 80. 33882874 10.1186/s12874-021-01268-z PMC8058580

[shil13431-bib-0034] Rai, T. , Morton, K. , Roman, C. , Doogue, R. , Rice, C. , Williams, M. , Schwartz, C. , Velardo, C. , Tarassenko, L. , Yardley, L. , Mcmanus, R. J. , & Hinton, L. (2021). Optimizing a digital intervention for managing blood pressure in stroke patients using a diverse sample: Integrating the person‐based approach and patient and public involvement. Health Expectations, 24, 327–340. 10.1111/hex.13173 33316120 PMC8077154

[shil13431-bib-0035] Rao, S. , Segar, M. W. , Bress, A. P. , Arora, P. , Vongpatanasin, W. , Agusala, V. , Essien, U. R. , Correa, A. , Morris, A. A. , De Lemos, J. A. , & Pandey, A. (2020). Association of genetic West African ancestry, blood pressure response to therapy, and cardiovascular risk among self‐reported black individuals in the Systolic Blood Pressure Reduction Intervention Trial (SPRINT). JAMA Cardiology, 6(4), 388–398. 10.1001/jamacardio.2020.6566. Epub ahead of print. PMID: 33185651; PMCID: PMC7666434. 33185651 PMC7666434

[shil13431-bib-0036] Roberts, D. E. (2021). Abolish race correction. The Lancet, 397, 17–18. 10.1016/S0140-6736(20)32716-1 33388099

[shil13431-bib-0037] Rooshenas, L. , Paramasivan, S. , Jepson, M. , & Donovan, J. L. (2019). Intensive triangulation of qualitative research and quantitative data to improve recruitment to randomized trials: The QuinteT approach. Qualitative Health Research, 29, 672–679. 30791819 10.1177/1049732319828693

[shil13431-bib-0038] Saini, A. (2019). Superior: The return of race science. Harper Collins.

[shil13431-bib-0039] Selvarajah, S. , Deivanayagam, T. A. , Lasco, G. , Scafe, S. , White, A. , Zembe‐Mkabile, W. , & Devakumar, D. (2020). Categorisation and minoritisation. BMJ Global Health, 5, e004508. 10.1136/bmjgh-2020-004508 PMC7780538 33380415

[shil13431-bib-0040] SHI (2020). SHI race and ethnicity research. Sociology of Health and Illness. https://onlinelibrary.wiley.com/doi/toc/ https://onlinelibrary.wiley.com/doi/toc/10.1002/1467‐9566.race‐ethnicity‐research

[shil13431-bib-0041] Smart, A. , & Weiner, K. (2018). Racialised prescribing: Enacting race/ethnicity in clinical practice guidelines and in accounts of clinical practice. Sociology of Health & Illness, 40, 843–858. 29626344 10.1111/1467-9566.12727 PMC6033176

[shil13431-bib-0042] The Stroke Association (2018). State of the nation: Stroke statistics. The Stroke Association.

[shil13431-bib-0043] Treweek, S. (2020). The forgotten, the ignored and the under‐served – trials cannot continue to fail large parts of society. http://blogs.biomedcentral.com/on‐medicine/2020/10/01/the‐forgotten‐the‐ignored‐and‐the‐under‐served‐trials‐cannot‐continue‐to‐fail‐large‐parts‐of‐society/

[shil13431-bib-0044] Wang, Y. , Rudd, A. G. , & Wolfe, C. D. (2013). Age and ethnic disparities in incidence of stroke over time: The South London Stroke Register. Stroke, 44, 3298–3304. 10.1161/STROKEAHA.113.002604 24114452

[shil13431-bib-0045] Washington, H. (2007). Medical apartheid: The dark history of medical experimentation on Black Americans from colonial times to the present. Doubleday.

[shil13431-bib-0046] Williams, D. R. , Lawrence, J. A. , Davis, B. A. , & Vu, C. (2019). Understanding how discrimination can affect health. Health Services Research, 54(Suppl. 2), 1374–1388. 31663121 10.1111/1475-6773.13222 PMC6864381

[shil13431-bib-0047] Williams, O. , Sarre, S. , Papoulias, S. C. , Knowles, S. , Robert, G. , Beresford, P. , Rose, D. , Carr, S. , Kaur, M. , & Palmer, V. J. (2020). Lost in the shadows: Reflections on the dark side of co‐production. Health Research Policy and Systems, 18, 43. 32380998 10.1186/s12961-020-00558-0 PMC7204208

[shil13431-bib-0048] Williams, R. (2021). “It's harder for the likes of us”: Racially minoritised stem cell donation as ethico‐racial imperative. BioSocieties, 16(4), 470–491. 10.1057/s41292-021-00241-9 34276806 PMC8275909

[shil13431-bib-0049] Wood, S. , Martin, U. , Gill, P. , Greenfield, S. M. , Haque, M. S. , Mant, J. , Mohammed, M. A. , Heer, G. , Johal, A. , Kaur, R. , Schwartz, C. , & Mcmanus, R. J. (2012). Blood pressure in different ethnic groups (BP‐Eth): A mixed methods study. British Medical Journal Open, 2, e001598. 10.1136/bmjopen-2012-001598 PMC3532997 23129572

[shil13431-bib-0050] Wright, N.‐M. (2020). BAME underrepresentation in clinical trials. British Journal of Cardiac Nursing, 15, 1–5.

[shil13431-bib-0051] Younis, T. , & Jadhav, S. (2020). Islamophobia in the National Health Service: An ethnography of institutional racism in PREVENT's counter‐radicalisation policy. Sociology of Health & Illness, 42, 610–626. 31849069 10.1111/1467-9566.13047

[shil13431-bib-0052] Yudell, M. , Roberts, D. , Desalle, R. , & Tishkoff, S. (2016). Taking race out of human genetics. Science, 351, 564–565. 26912690 10.1126/science.aac4951

